# Targeted Therapies and Biomarkers in Small Cell Lung Cancer

**DOI:** 10.3389/fonc.2020.00741

**Published:** 2020-05-20

**Authors:** Hirokazu Taniguchi, Triparna Sen, Charles M. Rudin

**Affiliations:** ^1^Molecular Pharmacology Program and Department of Medicine, Memorial Sloan Kettering Cancer Center, New York, NY, United States; ^2^Department of Respiratory Medicine, Nagasaki University Graduate School of Biomedical Sciences, Nagasaki, Japan; ^3^Department of Medicine, Weill Cornell Medical College, New York, NY, United States

**Keywords:** SCLC, targeted therapy, immune therapy, DNA damage repair pathway, biomarker

## Abstract

Small cell lung cancer (SCLC) is an aggressive malignancy characterized by rapid growth, early metastasis, and acquired therapeutic resistance. A majority of patients with SCLC have extensive-stage (ES) disease, defined as the presence of metastatic disease outside the hemithorax at first diagnosis. SCLC has been considered “a graveyard for drug development,” with chemotherapy remaining the standard treatment for first- and second-line management until quite recently. In contrast to NSCLC, identifying therapeutic targets in SCLC has been challenging, partly because driver mutations are primarily loss of function, involving the tumor suppressor genes RB1 and TP53 or currently untargetable (e.g., amplification of MYC family members). Recent gene expression profiling of SCLC cells lines, patient samples and representative murine models, have led to a proposed delineation of four major subtypes for SCLC distinguished by differential expression of four key transcriptional regulators (ASCL1, NEUROD1, POU2F3, and YAP1). Our understanding of the biology of SCLC has indeed significantly improved recently due to the continued efforts of the dedicated investigators in this field, but the therapeutic options remain dismal. While recent results from immunotherapy trials are encouraging, most patients demonstrate either primary or rapid acquired resistance to current regimens, highlighting the clear need to improve the effectiveness and expand the scope of current therapeutic strategies. In this opinion article, we will discuss recent developments in the treatment of SCLC, focused on current understanding of the signaling pathways, the role of immunotherapy and targeted therapy, and emerging biomarkers of response to therapy in SCLC.

## Introduction

**Figure 1:**
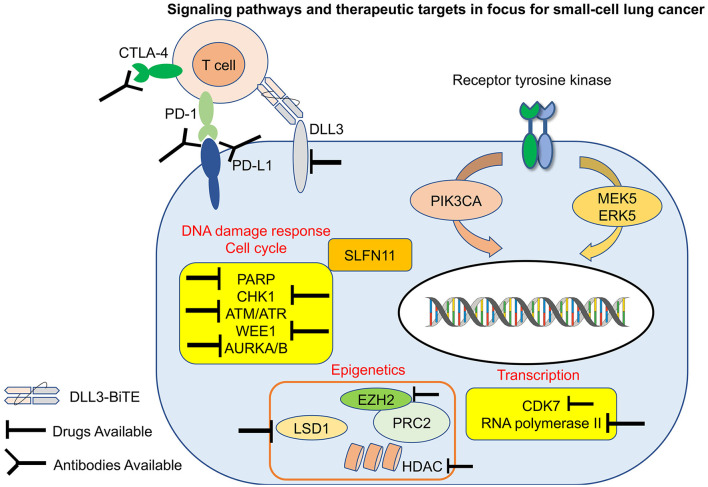
Signaling pathways and therapeutic targets in focus for small-cell lung cancer (SCLC). Notable targets and evolving treatment strategies in SCLC including immunotherapy, targeted therapy, antibody drug conjugates. PD-1, programmed death-1; PD-L1, programmed death ligand-1; CTLA-4, cytotoxic T lymphocyte associated protein 4; DLL3, delta-like 3; PIK3CA, phosphatidylinositol-4,5-bisphosphate 3-kinase catalytic subunit alpha; AURKA/B, aurora kinase A/B; CHK1, checkpoint kinase 1; PARP1, poly-ADP ribose polymerase 1; EZH2, enhancer of zeste 2; LSD1, lysine-specific demethylase 1A; HDAC, histone deacetylase; ATR, ataxia telangiectasia and RAD3-related protein; ATM, ataxia telangiectasia mutated; PRC2, polycomb repressor complex 2; CDK7, cyclin-dependent kinase 7; SLFN11, schlafen11.

Small cell lung cancer (SCLC) is an aggressive malignancy characterized by rapid growth, early metastasis, and acquired therapeutic resistance (1–3). A majority of patients with SCLC have extensive-stage (ES) disease, defined as the presence of metastatic disease outside the hemithorax at first diagnosis. Although the clinical treatment for non-small cell lung cancer (NSCLC) has changed dramatically and rapidly, SCLC has been considered “a graveyard for drug development,” with chemotherapy remaining the standard treatment for first- and second-line management for over four decades until quite recently. In contrast to NSCLC, identifying therapeutic targets in SCLC has been challenging, partly because driver mutations are primarily loss of function, involving the tumor suppressor genes *RB1* and *TP53* (4, 5) or currently untargetable (e.g., amplification of *MYC* family members). Recent gene expression profiling of SCLC cells lines, patient samples and representative murine models, have led to a proposed delineation of four major subtypes for SCLC distinguished by differential expression of four key transcriptional regulators (ASCL1, NEUROD1, POU2F3, and YAP1). Our understanding of the biology of SCLC has indeed significantly improved recently due to the continued efforts of the dedicated investigators in this field, but the therapeutic options remain dismal. While recent results from immunotherapy trials are encouraging, most patients demonstrate either primary or rapid acquired resistance to current regimens, highlighting the clear need to improve the effectiveness and expand the scope of current therapeutic strategies. In this opinion article, we will discuss recent developments in the treatment of SCLC, focused on current understanding of the signaling pathways, the role of immunotherapy and targeted therapy, and emerging biomarkers of response to therapy in SCLC (Figure 1).

## New Therapeutic Targets for SCLC

SCLC tumors typically carry a high mutation burden and have evident genomic instability manifest by aneuploidy and multiple intra- and inter-chromosomal rearrangements. Almost all SCLC tumors have functional inactivation of both *TP53* and *RB1*; however, attempts to target these genomic alterations have resoundingly failed. Recent studies using comprehensive whole exome and whole genome sequencing in SCLC have revealed that SCLC tumors include other recurrent genomic alternations (4–6). Visualization of the SCLC genomic landscape has led to the identification of new targets such as *PTEN* loss (7), activating *PI3K* mutations (8, 9), and *FGFR1* amplifications (10, 11). The novel therapeutic targets, corresponding drugs and the predictive biomarkers were summarized in Table 1.

**Table 1:** Novel treatment targets and the corresponding drugs, predictive biomarkers in SCLC.

**Targets**	**Drug**	**Biomarker**
PARP	Olaparib	SLFN11
	Veliparib	
	Talazoparib	
	Niraparib	
	Rucaparib	
CHK1	Prexasertib	MYC
	PF-477736	
	MK-8776	
	GDC-0575	
	SRA737	
ATM/ATR	VX-970	NA
	VX-803	
	AZD6738	
WEE1	AZD1775	NA
AURKA/B	Barasertib	MYC
	Alisertib	
PD-1	Nivolumab	Tumor mutation burden
	Pembrolizumab	
PD-L1	Atezolizumab	NA
	Durvalumab	
CTLA-4	Ipilimumab	Tumor mutation burden
DLL3	Rova-T	DLL3
	AMG 757	
	AMG 119	
FGFR	Ponatinib	NA
	Lucitanib	
EZH2	DS-3201b	NA
	Tazemetostat	
LSD1	GSK2879552	NA
	T-3775440	
CDK7	YKL-5-124	NA
RNA polymerase II	Lurbinectedin	NA

### DNA Damage Repair Pathway and Cell Cycle

The high mutation burden of SCLC is largely attributable to the strong association of this disease with heavy tobacco exposure, with only 2% of cases occurring in never smokers (12–14). The loss of cell cycle checkpoint controls due to inactivation of RB1 and TP53 may increase susceptibility of SCLC to DNA damage. Indeed, multiple reports in the past few years have convincingly pointed to DNA damage response (DDR) pathways as critical vulnerabilities in SCLC. Targeting central DDR mediators, such as poly ADP-ribose polymerase (PARP), checkpoint kinase 1 (CHK1), Ataxia telangiectasia and RAD3-related protein (ATR), Ataxia telangiectasia mutated (ATM), and WEE1, have demonstrated promising therapeutic opportunities in SCLC.

The anti-tumor activities of PARP inhibitors occur through multiple mechanisms, including (1) trapping the enzyme to the single-strand DNA breaks (SSBs) by preventing the utilization of nicotinamide adenine dinucleotide (NAD), and (2) inhibiting poly ADP-ribosylation (PARylation) and binding of PARP to DNA (15). The PARP inhibitor AZD2281 was found to have greater against SCLC cell lines than NSCLCs (16). PARP inhibitors with PARP trapping activity sensitized SCLC cell lines and patient-derived xenografts to ionizing radiation (17). A phase 1 trial demonstrated initial promising activity of the potent PARP trapping drug talazoparib, including in patients with SCLC (18).

SCLC cell lines have a higher median CHK1 protein and gene expression than NSCLC lines, and the CHK1 inhibitor prexasertib demonstrated strong anti-tumor activity in SCLC cell lines, SCLC syngeneic, genetically-engineered mouse (GEM) and chemo-resistant models (19). The effectiveness of targeting CHK1/ATR axis in SCLC was later confirmed in an independent preclinical study with ATR inhibitors in particular demonstrating activity against SCLC in both *in vitro* and *in vivo* models (20). Activation of ATR through DNA damage stimulates multiple downstream targets including CHK1, which halts cell cycle progression at the G2-M phase (21, 22). The G2/M checkpoint regulator WEE1 is also upregulated in SCLC cell lines relative to normal lung tissue or NSCLCs, and the WEE1 inhibitor AZD1775 showed activity in several SCLC cell lines (23).

Inhibition of Aurora kinase A or B inhibits the proliferation, growth of SCLC *in vitro* and *in vivo* (24, 25). A recently reported clinical trial demonstrated that the aurora kinase A inhibitor alisertib plus paclitaxel had significantly improved PFS vs. paclitaxel alone in patients with cMYC positive SCLC (26).

Finally, several preclinical and clinical trials have demonstrated that combining DDR inhibitors with chemotherapy or other targeted agents could be a promising strategy (16, 23, 27–31).

### Targeting Epigenetic Modifiers in SCLC

Visualizing the human epigenome using next generation sequencing highlighted the role of epigenetic processes in cancer generally, and SCLC in particular (32–34). Here we focus on two of the most promising epigenetic regulatory proteins; enhancer of zeste homology 2 (EZH2) and lysine-specific demethylase 1A (LSD1), both of which are now being tested in current and upcoming SCLC clinical trials.

EZH2 is one of the enzymatic histone-lysine N-methyltransferase subunits of polycomb repressor complex 2 (PRC2), which primarily inhibits gene expression by promoting tri-methylation of Histone 3 on lysine at position 27. EZH2 expression is higher in SCLC than in any tumor type included in the Cancer Genome Atlas (34), and preclinical analysis showed that an EZH2 inhibitor augmented chemotherapeutic efficacy and could prevent emergence of acquired chemotherapy resistance in multiple *in vivo* SCLC patient-derived xenograft models (35). A phase I/II study to test this strategy in clinic has been launched, using the EZH1/2 inhibitor DS-3201b together with irinotecan in patients with recurrent SCLC (NCT03879798). Further raising interest in EZH2 as a target in SCLC, it has been recently demonstrated that PRC2 transcriptionally suppresses MHC class I expression in SCLC, suggesting that EZH2 inhibition may also augment SCLC response to immune check point inhibitors (36).

LSD1, a monoamine oxidase that demethylates mono- or di-methylated lysine 4 or lysine 9 of histone H3, has been implicated in oncogenesis and depending on context can either activate or repress gene transcription (37). LSD1 inhibitors has been shown to exert anticancer effects against SCLC *in vitro* and *in vivo* through inhibition of the interaction between LSD1 and SNAG domain proteins; insulinoma-associated protein 1 (INSM1) or Growth factor independence 1B (38, 39).

### Immunotherapy Regimens and Combinations for SCLC

Immunotherapy using checkpoint inhibitory monoclonal antibodies blocking programmed cell death 1 (PD-1), programmed death-ligand 1 (PD-L1), and cytotoxic T-lymphocyte-associated protein 4 (CTLA-4), either as single agents or in combination, have led to a revolution in the treatment of several solid tumors, including NSCLC. Inhibition of these immune checkpoint molecules can prompt reactivation of cytotoxic T cell immunity that had been held in check, in some cases resulting in durable anticancer responses even in patients with advanced disease.

Recently, the United States Food and Drug Administration approved the PD-1 inhibitors, nivolumab and pembrolizumab, as the third line treatment options for SCLC patients. The phase 1/2 CheckMate 032 trial, which explored the efficacy of nivolumab alone or in combination with two different doses of ipilimumab, and the phase 1b KEYNOTE-028 and phase 2 KEYNOTE-158 trials, which examined the efficacy of pembrolizumab for pretreated patients with SCLC with PD-L1 positive tumors, demonstrated efficacy in previously treated SCLC patients (40–42). Unfortunately, while response rates on these trials ranged from 11 to 33%, most patients treated did not appear to benefit, as evidenced by median progression-free survivals of only 1.4–2 months. A small number of patients, <10%, demonstrate long-term responses on these trials, prompting intensive and ongoing investigation into biomarkers that might discriminate these patients, and exploration of combination therapies that might increase the fraction of patients with durable benefit.

A pair of recent landmark studies in SCLC have explored the efficacy of PD-L1 inhibitors combined with cytotoxic agents for newly diagnosed extensive stage patients. In the first line setting, the PD-L1 inhibitor atezolizumab combined with carboplatin plus etoposide was approved by the FDA based on the results of the IMPOWER133 study (43). Similarly, an initial report from the CASPIAN trial demonstrated that first-line durvalumab plus platinum–etoposide also significantly improved OS in patients with ES-SCLC (44). These studies established the benefit of adding a PD-L1 inhibitor to platinum plus etoposide and confirmed the promise of immune check point inhibitors for the treatment of patients with SCLC. Notably a third trial, KEYNOTE-604, assessing the addition of pembrolizumab to first line carboplatin and etoposide, demonstrated a similar improvement in PFS to the studies of the PD-L1 inhibitors above, but narrowly missed statistical significance for an OS benefit; detailed results of this study have not yet been presented or published.

Building on these initial important but limited successes, ongoing preclinical and clinical studies are now exploring combined therapies with PD-1 or PD-L1 inhibitors and compounds which target epigenetic modifiers, DDR proteins, or cell cycle regulators. Some of these have already shown promising results for SCLC in preclinical studies. As noted above, treatment with an EZH2 inhibitor can upregulate MHC class I expression, promoting better antigenic presentation by tumor cells and significant tumor suppression when combined with immune checkpoint inhibitors (36). Furthermore, the combination of a CHK1 inhibitor and low dose gemcitabine enhanced the effect of PD-L1 inhibition (45), and concomitant treatment with a DDR inhibitor remarkably potentiated the antitumor effects of PD-L1 inhibition in mouse models of SCLC (46). Treatment with a cyclin-dependent kinase 7 inhibitor, YKL-5-124, was also found to enhance anti-tumor efficacy of a PD-1 inhibitor in SCLC preclinical models (47). Several clinical trials using immune checkpoint inhibitor combinations are ongoing (48).

### Other Emerging Targets in SCLC

Genomic profiling of SCLC has demonstrated common inactivating mutations in the primary Notch family members (4) and aberrant overexpression of a key negative regulator of Notch signaling, delta-like protein 3 (DLL3) was found in the majority of SCLC tumors (49). Initial clinical evaluation of an anti-DLL3 antibody-drug conjugate rovalpituzumab teserine (Rova-T) demonstrated promising activity, although the ultimate utility of this agent was compromised by toxicities (50, 51). DLL3 remains a target of substantial interest for drug development in SCLC, with active strategies including an anti-DLL3/anti-CD3 bispecific T-cell engager (NCT03319940).

The association of SCLC with selective activation of master transcriptional regulators has raised interest in strategies focused on modulation of transcriptional control. Lurbinectidin, a DNA binding agent that appears to function as a selective inhibitor of RNA polymerase II transcription, has demonstrated substantial activity against SCLC both as a single agent and in combination with doxorubicin (52–54).

Other targets of recently emerging interest include selective metabolomic dependencies of SCLC. MEK5 and ERK5 have been recently identified as critical regulators of lipid metabolism of SCLC cells, suggesting these kinases as possible therapeutic targets (55). MYC-driven SCLC cells have been reported to be highly dependent on arginine-regulated pathways including polyamine biosynthesis and mTOR pathway activation; selective arginine depletion appeared to be highly effective in MYC-driven SCLC preclinical models (56).

## Biomarkers in SCLC

### Biomarkers of Targeted Therapy

Biomarkers predictive of response to the therapy are urgently needed to guide treatment selection for patients with SCLC. Studies from multiple groups have suggested that Schlafen11 (SLFN11) expression is a potential biomarker of sensitivity of both DNA damaging chemotherapy and PARP inhibition (29, 35, 57, 58). Genetic alteration of *MYC*, most commonly gene amplification, was observed in approximately 20% of SCLC, placing it among the most common genetic abnormalities after *TP53* and *RB1*. Higher expression or amplification of *MYC* predicted sensitivity to CHK1 inhibition in SCLC (19, 59, 60) and Aurora Kinase inhibition (61). High expression of DLL3 on cell surface of SCLC was associated with better response to Rova-T in both preclinical and clinical studies (49–51).

### Biomarkers of Immunotherapy

Expression of PD-L1 on tumor cells has been shown to be an enrichment factor for efficacy of PD-1 inhibition in many solid tumors including NSCLC (62, 63). The predictive value of PD-L1 expression for the treatment of SCLC with PD-1 inhibitors is currently unknown. Challenging the utility of PD-L1 expression as a useful biomarker in SCLC is a lower prevalence, heterogeneous expression on SCLC tumors, and the lack of clear correlation between PD-L1 expression and the effect of immunotherapy (40, 41). In contrast to the expression of PD-L1, a detailed retrospective biomarker analysis of patients enrolled in the CheckMate 032 trial suggested improved ORR, OS, and PFS of nivolumab monotherapy or nivolumab plus ipilimumab combination therapy in patients with a high tumor mutation burden (TMB) relative to patients with a low/medium TMB (64).

The presence of a high number of tumor-infiltrating lymphocytes (TILs), mismatch repair (MMR) deficiency, or a high frequency of microsatellite instability (MSI) also predicted improved response to immune checkpoint inhibitors in other types of cancers (65–69). The recent phase II clinical trial, KEYNOTE-158, demonstrated the clinical benefit of therapy with pembrolizumab among patients with previously treated unresectable or metastatic, high MSI, DNA MMR deficient, non-colorectal cancer (70); with only four patients with SCLC enrolled in this study, further investigation will be required.

## Future Strategies for SCLC

As in other solid tumors, therapies directed toward induction of anti-tumor immunity are likely to play an increasingly important role in the treatment of SCLC. Immune checkpoint inhibitors have shown modest yet promising effects when combined with platinum plus etoposide for patients with ES SCLC. However, continued efforts need to be made to achieve more durable and more broadly effective immunotherapy responses in SCLC. Future research efforts should seek to identify not only effective combinatorial regimens, but also predictive markers of immunotherapy in SCLC. Another area of current intensive investigation is the analysis of SCLC subtype-specific therapeutic vulnerabilities and predictive biomarkers associated with particular treatment outcomes for each of the four major subtypes. Recent insights into the biology of SCLC have promoted the development of molecular targeted and immunologic strategies for what has been a particularly refractory disease. Future research and improved clinical prospects for patients with SCLC will depend on continued focus on an integrated platform of basic discovery and clinical translational research, identifying novel biomarker-driven approaches to integrate immunotherapy and other targeted therapies.

## Author Contributions

HT, TS, and CR contributed to write the manuscript.

## Conflict of Interest

CR has previously consulted for AbbVie, Amgen, Ascentage, Astra Zeneca, Celgene, Daiichi Sankyo, Genentech/Roche, Ipsen, Loxo, PharmaMar, Harpoon Therapeutics, and Bridge Medicines. The remaining authors declare that the research was conducted in the absence of any commercial or financial relationships that could be construed as a potential conflict of interest.
